# MSXFGP: combining improved sparrow search algorithm with XGBoost for enhanced genomic prediction

**DOI:** 10.1186/s12859-023-05514-7

**Published:** 2023-10-11

**Authors:** Ganghui Zhou, Jing Gao, Dongshi Zuo, Jin Li, Rui Li

**Affiliations:** 1 College of Computer and Information Engineering, Inner Mongolia Agricultural University, Erdos East Street No. 29, Hohhot, 010011 China; 2 Inner Mongolia Autonomous Region Key Laboratory of Big Data Research and Application for Agriculture and Animal Husbandry, Zhaowuda Road No. 306, Hohhot, 010018 China; 3 Inner Mongolia Autonomous Region Big Data Center, Chilechuan Street No. 1, Hohhot, 010091 China

**Keywords:** Genome selection, Sparrow search algorithm, XGBoost, Parameter optimization, Feature selection

## Abstract

**Background:**

With the significant reduction in the cost of high-throughput sequencing technology, genomic selection technology has been rapidly developed in the field of plant breeding. Although numerous genomic selection methods have been proposed by researchers, the existing genomic selection methods still face the problem of poor prediction accuracy in practical applications.

**Results:**

This paper proposes a genome prediction method MSXFGP based on a multi-strategy improved sparrow search algorithm (SSA) to optimize XGBoost parameters and feature selection. Firstly, logistic chaos mapping, elite learning, adaptive parameter adjustment, Levy flight, and an early stop strategy are incorporated into the SSA. This integration serves to enhance the global and local search capabilities of the algorithm, thereby improving its convergence accuracy and stability. Subsequently, the improved SSA is utilized to concurrently optimize XGBoost parameters and feature selection, leading to the establishment of a new genomic selection method, MSXFGP. Utilizing both the coefficient of determination R^2^ and the Pearson correlation coefficient as evaluation metrics, MSXFGP was evaluated against six existing genomic selection models across six datasets. The findings reveal that MSXFGP prediction accuracy is comparable or better than existing widely used genomic selection methods, and it exhibits better accuracy when R^2^ is utilized as an assessment metric. Additionally, this research provides a user-friendly Python utility designed to aid breeders in the effective application of this innovative method. MSXFGP is accessible at https://github.com/DIBreeding/MSXFGP.

**Conclusions:**

The experimental results show that the prediction accuracy of MSXFGP is comparable or better than existing genome selection methods, providing a new approach for plant genome selection.

## Background

Genomic Selection (GS), also known as genomic prediction, is a breeding method based on genome-wide marker information, a concept introduced by Professor THE Meuwissen of the Norwegian University of Life Sciences in 2001 [1]. It is widely used in animal and plant breeding for early individual prediction and selection based on genomic estimated breeding values, thus enabling the reduction of generational intervals.

As research has progressed, scientists have proposed numerous different Genomic Selection (GS) methodologies. Among these, Best Linear Unbiased Prediction (BLUP) [2] is a widely adopted approach. It leverages pedigree information to define the genetic relationship matrix among individuals, thereby providing unbiased estimates of individual breeding values. Genomic BLUP (GBLUP) [3] extends BLUP by using genome-wide genetic markers to construct a genetic relationship matrix, thereby predicting missing phenotypic values. Ridge Regression BLUP (RR-BLUP) [4], on the other hand, uses shrinkage estimation to capture marker effects for genome-wide prediction, but it may suffer from over-shrinkage issues. BayesA [5] and BayesB [6] methods remedy the shortcomings of RR-BLUP [7] by positing that each marker effect follows a prior distribution, with BayesB's prior being a mixture distribution. In addition, there are also methods such as BayesC [8] and Bayesian LASSO [9].

However, most of the above-mentioned GS methods are based on compression estimation methods, and their calculation speed will be limited as the data dimension increases [10], which is not suitable for the massive genetic marker data generated by modern sequencing technologies. Therefore, researchers began to continuously explore the use of machine learning and deep learning methods. For example, some machine learning algorithms, such as Support Vector Regression (SVR), Random Forest (RF), and LightGBM, have been successfully applied to genome selection [11]. These methods can handle high-dimensional data and can capture complex nonlinear relationships, improving the accuracy of genomic selection. In addition, deep learning methods such as DeepGS [12] and DNNGP [13] also provide new possibilities for genome selection. Deep learning models have the ability to process massive data and automatically extract complex features, which can further improve the prediction accuracy of genome selection.

Although scientists have proposed many different genome selection (GS) methods, in practical applications, the prediction accuracy of existing genome selection methods is often low [14], mainly because machine learning and deep learning models need to rely on rich experience in hyperparameter tuning [15], which largely limits its effectiveness in practical applications. In addition, the number of genotype data markers is often far greater than the number of samples [16], which makes overfitting prone to occur in the development of predictive models. Overfitting models may exaggerate small fluctuations in the data, resulting in poorer predictive power in the end. Although there are also many processing methods to address high-dimensional data, such as principal component analysis (PCA) [17] and factor analysis (FA) [18], these methods often result in the loss of some important information from the data, which affects the accuracy of prediction. The complexity of adjusting hyperparameters in machine learning methods and the redundant information in high-dimensional features hinder the further application of machine learning in genomic selection.

However, metaheuristic algorithms and swarm intelligence algorithms, as two powerful optimization methods, have received extensive attention and research in several research areas in recent years. These two types of optimization algorithms aim to draw inspiration from natural phenomena or biological processes to solve complex optimization problems and find the best or approximate solutions to these problems. They have been successfully applied to key issues like the traveling salesman problem (TSP) [19], parameter optimization, and feature selection. For instance, many researchers have effectively utilized optimization algorithms such as the Genetic Algorithm (GA), Ant Colony Optimization (ACO), Bee Colony Optimization (BCO), and Particle Swarm Optimization (PSO) to address the TSP problem [20]. Furthermore, many researchers have applied optimization algorithms to the optimization of model parameters and feature selection. For example, Elnaz Pashaei [21] combined the swarm intelligence algorithm COOT with the metaheuristic algorithm Simulated Annealing for feature selection in high-dimensional microarray data, enhancing the experimental outcomes. Elham Pashaei [22] employed an improved Black Hole Algorithm (BHA) to find the optimal weights and biases for Feedforward Neural Networks (FNN), increasing the model’s accuracy. Moreover, some researchers have tried to use optimization algorithms for simultaneous feature selection and parameter optimization to enhance model results. A salient example of this is the work by Zhou Tao [23], who proposed a feature selection and parameter optimization method for Support Vector Machines (SVM) based on the Genetic Algorithm (GA). This method can quickly obtain suitable feature subsets and SVM parameters, yielding better results.

The sparrow search algorithm (SSA) used in this paper is a new type of swarm intelligence algorithm inspired by the foraging and anti-predatory behavior of sparrows. This algorithm exhibits superiority in terms of convergence speed, search accuracy, and stability [24]. As a result, SSA has been extensively applied to various optimization problems, such as feature selection, energy consumption, scheduling, and engineering problems [25]. However, it still has some limitations that require further improvement to better address real-world issues. Therefore, inspired by previous research, this paper introduces multiple strategic improvements to SSA and combines it with XGBoost [26] achieving simultaneous parameter tuning and feature selection, thus constructing a new genome prediction method MSXFGP. In addition, to ensure the reliability of the method, we use the coefficient of determination R2 [27] and Pearson correlation coefficient [28] as evaluation indexes at the same time and analyze the potential defects of using only the Pearson correlation coefficient as evaluation indexes in our experiments. In this study, we selected six datasets of five crops to compare and analyze with six existing genome selection methods and verified the accuracy of MSXFGP for crop genome prediction. Finally, in order to make the model more practical, we provide users with an easy-to-use tool based on Python, which will significantly reduce the threshold for breeding professionals to use this model, and further promote its application in actual breeding work.

## Materials and methods

### Datasets and pre-processing

Five different crops including japonica, groundnut, wheat, tomato and potato were selected for this study, where two different-sized datasets were selected for potato. The following provides a detailed description of the genotype and phenotype data for each dataset.

#### Japonica

Japonica was studied by Monteverde et al. [29, 30]. The genotype data was obtained using Genotyping-by-Sequencing (GBS), and missing data was processed and filled using the FILLIN algorithm in TASSEL 5.0. This resulted in a total of 320 sequenced samples, each containing 44,598 markers. For the convenience of statistical analysis, the genotype data was converted into numerical codes of 0, 1, and 2. Phenotype data for grain yield (GY) traits from 2012 was selected. This phenotype data is real and has not been processed in any way. After merging the phenotype data with the genotype samples, a total of 316 samples were selected that had both genotype and phenotype data.

#### Groundnut

Pandey and his team used the Affymetrix GeneTitan platform to extract the genomic information of 318 lines of Groundnut and perform SNP genotyping [29, 31]. After quality control, each genotype contained 8268 SNP markers, which were coded as 0, 1, and 2. The selected phenotype data were the yield per hectare (YPH) trait from the ALIYARNAGAR_R15 environment.

#### Wheat

This dataset was collected by the Global Wheat Program of the International Maize and Wheat Improvement Center (CIMMYT) [13]. They utilized Diversity Arrays Technology (DArT) markers for genotyping, which were recorded in the form of presence (1) or absence (0) [32]. To ensure data quality, markers with minor allele frequencies less than 0.05 were removed. After quality control, the data contained 1279 markers. The phenotype data chosen came from grain yield (GY) in the "env1" environment, with a total of 599 samples. The phenotype data underwent standardization, transforming it into a distribution with a mean of 0 and a standard deviation of 1.

#### Tomato

Yao Zhou et al. [33] first mapped the Illumina sequences of 332 tomato germplasms to TGG1.1 and genotyped the genetic variants of the tomato, resulting in 6,971,059 SNPs. Kelin Wang [13] then applied PCA (Principal Component Analysis) for dimensionality reduction, ultimately obtaining genotype data with 251 features. The phenotype data is the data of the fruit soluble solid content (SSC) traits of the 332 samples, which were transformed by log10 by the original author.

#### Potato1

This dataset, comprising 2500 markers, was genotyped using targeted gene sequencing methods carried out by AgriTech-Intertek ScanBi Diagnostics in Alnarp, Sweden [34]. The genotype data was encoded as 0, 1, 2, 3, and 4, representing the tetraploid alleles AAAA, AAAB, AABB, ABBB, and BBBB respectively. The phenotype data selected was for the total tuber yield trait under the Mosslunda environment, with a total of 253 samples.

#### Potato2

This dataset was genotyped using the GGPv3.0 array [35]. After quality control, SNP markers with minor allele frequencies less than 0.05 were discarded, resulting in a total of 10,546 markers. The genotype data is numerical, consisting of five allelic states ranging from 0 to 4. Here, 0 and 4 represent two homozygotes OOOO and AAAA, while 1, 2, and 3 correspond to the three heterozygotes AOOO, AAOO, and AAAO respectively. The selected phenotype data were averages of the tuber weight trait at various locations over many years, with a total of 669 samples.

### XGBoost

XGBoost is a machine learning algorithm based on Gradient Boosted Decision Trees (GBDT), proposed by Tianqi Chen in 2016 [26], the core of the algorithm lies in gradient boosting, which forms a powerful prediction model by integrating a large number of weak learners (generally decision trees), and each new weak learner is trained based on the residuals of the prediction of the previous learners, thus continuously reducing the prediction error of the model in each iteration. This gradient boosting-based approach results in excellent prediction performance of XGBoost. Because of its excellent performance and efficient computing power, XGBoost is favored in many data science competitions and practices in various fields and is often selected as the preferred model. Therefore, this study also selects XGBoost model for genome selection.

### SSA

Sparrow search algorithm (SSA) [24] is a swarm intelligence optimization algorithm [36] that optimizes the search goal by mimicking the foraging and antipredator behaviors in a sparrow population. It uses three types of sparrow foraging behaviors: discoverer, follower and vigilante to perform goal finding to iteratively optimize the search.

First of all, this algorithm is similar to most swarm intelligence optimization algorithms. It needs to initialize a population $X$ according to the population dimension $Dim$, the number of sparrows $N$, the upper bound $ub$ of the solution space, and the lower bound $lb$ of the solution space, as shown in formula (1):

1 $$X = \left[ {\begin{array}{*{20}c} {x_{1,1} } & {x_{1,2} } & {...} & {x_{1,j} } \\ {x_{2,1} } & {x_{2,2} } & {...} & {x_{2,j} } \\ \vdots & \vdots & \vdots & \vdots \\ {x_{i,1} } & {x_{i,2} } & {...} & {x_{i,j} } \\ \end{array} } \right]$$

Among them, $X_{i,j}$ is the position of the sparrow, $i = 1,2, \ldots ,N$, $j = 1,2, \ldots ,Dim$, and $lb_{j} \le X_{i,j} \le ub_{j}$, where the population dimension $Dim$ is also the length of the solution space.

In SSA, the discoverer tends to prioritize access to food based on its own higher fitness and leads the group to forage. The position update strategy is shown in formula (2):

2 $$X_{i,j}^{t + 1} = \left\{ \begin{aligned} X_{i,j}^{t} \cdot \exp \left( {\frac{ - i}{{\alpha \cdot M}}} \right),R_{{}} < ST \hfill \\ X_{i,j}^{t} + Q \cdot L,R_{{}} > ST \hfill \\ \end{aligned} \right.$$

Among them, $t$ is the current number of iterations, $j$ is the number of dimensions, and $X_{i,j}$ represents the position information of the $i$ sparrow in the $j$ dimension. $M$ is the maximum number of iterations. ${\upalpha } \in {(0,1]}$ is a random number. ${\text{R}},{\text{ST}}$ are the early warning value and the alert value respectively, ${\text{R}}$ is a random number within the range [0,1], and ${\text{ST}} \in {[0}{\text{.5,1]}}$ is a user-defined value. ${\text{Q}}$ is a random number that obeys a normal distribution. ${\text{L}}$ is a ${1} \times {\text{d}}$ identity matrix. When ${\text{R}} < {\text{ST}}$, it means that there are no predators around the foraging environment, and individual sparrows can perform extensive search operations. If ${\text{R}} \ge {\text{ST}}$, it means that the sparrows in the population have discovered the predator. At this time, it is necessary for the discoverer to lead the sparrow population to fly to other safe places for food.

The follower will follow the discoverer's lead to perform foraging behaviour, and the follower position update formula is:

3 $$X_{i,j}^{t + 1} = \left\{ \begin{aligned} Q \cdot \exp \left( {\frac{{X_{worst} - X_{i,j}^{t} }}{{i^{2} }}} \right),\quad i > n/2 \hfill \\ X_{P}^{t + 1} + \left| {X_{i,j}^{t} - X_{P}^{t + 1} } \right| \cdot A^{ + } \cdot L,\quad i \le n/2 \hfill \\ \end{aligned} \right.$$

Among them, $X_{p}$ is the optimal position currently occupied by the discoverer and $X_{{{\text{worst}}}}$ denotes the current global worst position. $A$ denotes a ${1} \times {\text{d }}$ matrix where each element is randomly assigned a value of 1 or − 1 and $A^{ + } = A^{T} (AA^{T} )^{ - 1}$. When ${\text{i}} > {\text{n/2 }}$, this indicates that the $i$ follower with a lower fitness value is not getting food and is in a very hungry state, and at this time needs to fly elsewhere to forage for food to get more energy.

Sparrows in the foraging process, some of the sparrows need to ensure the safety of the whole group of the problem, to guide the group to avoid predators, such sparrows are known as vigilante, and their position update formula is

4 $$X_{i,j}^{t + 1} = \left\{ \begin{aligned} X_{best}^{t} + \beta \left| {X_{i,j}^{t} - X_{best}^{t} } \right|,\quad f_{i} \ne f_{g} \hfill \\ X_{best}^{t} + k\left( {\frac{{X_{i,j}^{t} - X_{best}^{t} }}{{\left| {f_{i} - f_{w} } \right| + \varepsilon }}} \right),\quad f_{i} = f_{g} \hfill \\ \end{aligned} \right.$$

Among them, $X_{{{\text{best}}}}$ is the current global optimal position. $\beta \sim {\text{N(0,1)}}$ is used as a step control parameter. ${\text{k}} \in {[} - {1,1]}$ is a random number, and $f_{i}$ is the fitness value of the current individual sparrow. $f_{g}$ and $f_{w}$ are the current global optimal and worst fitness values, respectively. $\varepsilon$ is a small constant to avoid the denominator being zero. When $f_{i} \ne f_{g}$, it means that this sparrow needs to change its position to make the fitness converge to the optimal fitness; when $f_{i} = f_{g}$, it means that this sparrow will be moving towards the better sparrow group.

### Multi-strategy improved SSA (MSSA)

**Fig. 1:**
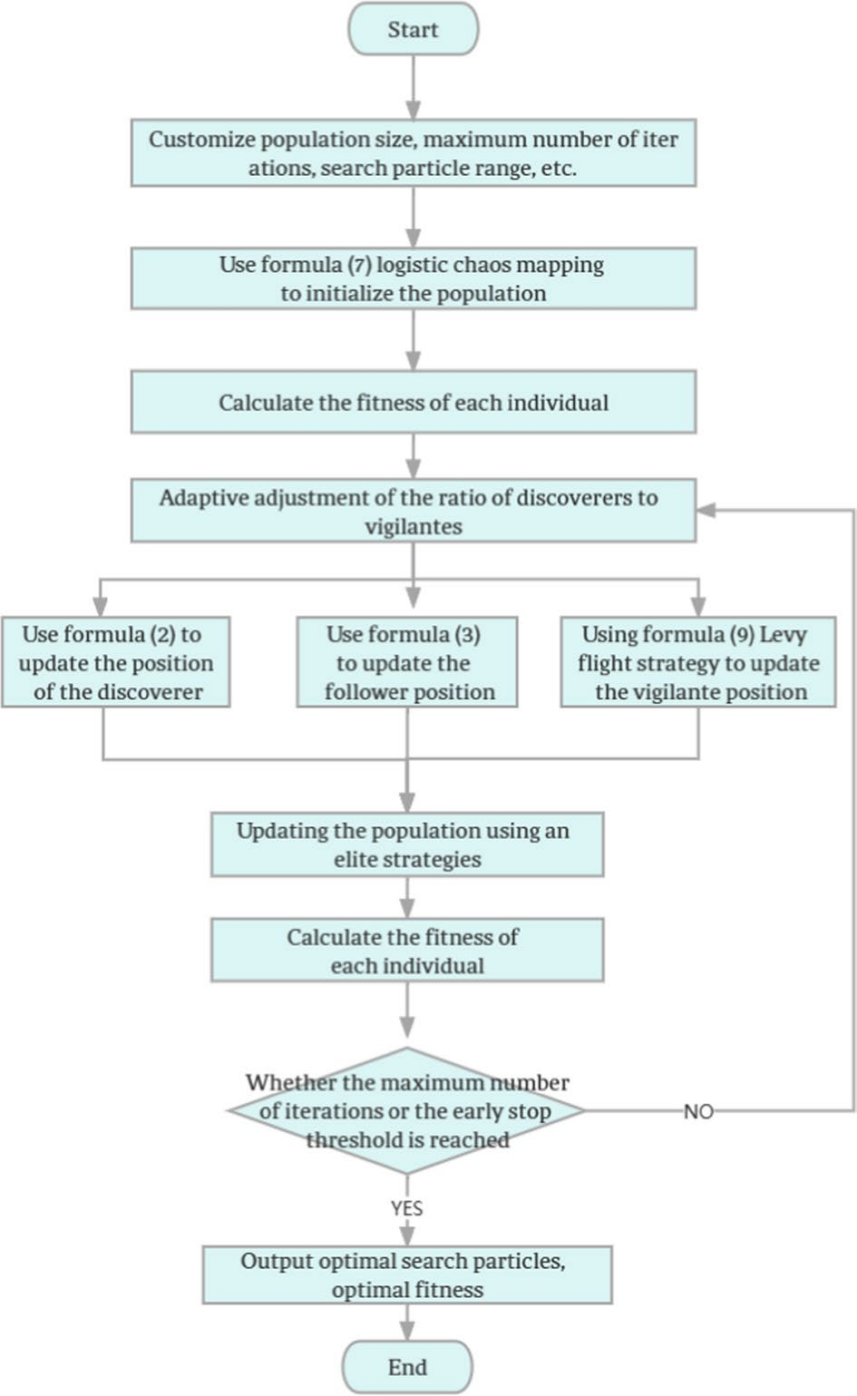
Flowchart of MSSA

**Fig. 2:**
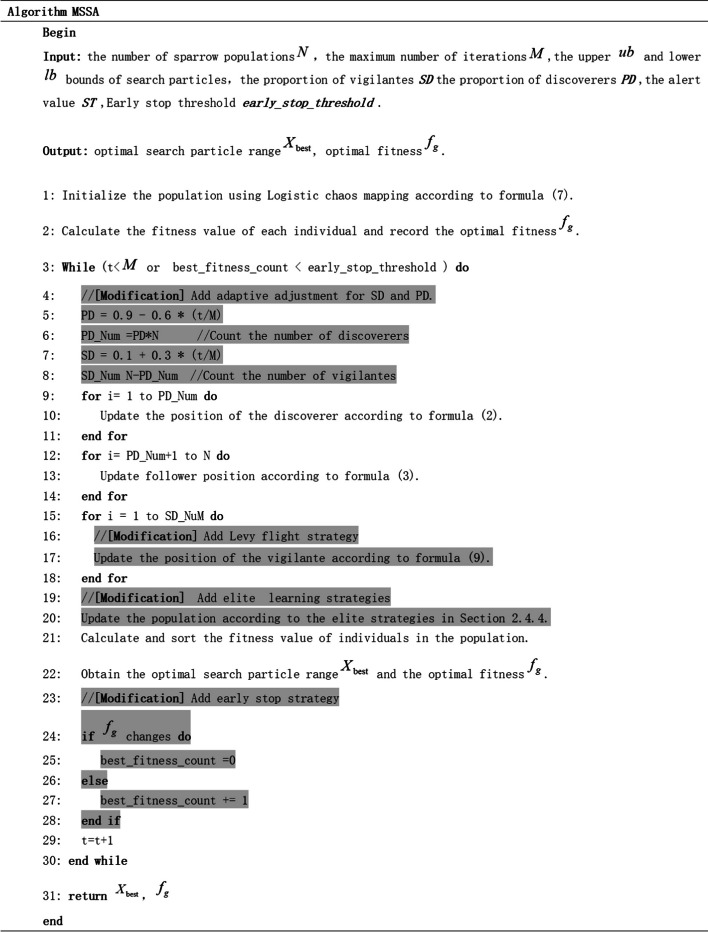
Pseudo-code of MSSA

The standard SSA still has some shortcomings, such as easy to fall into local optimum and low accuracy in the later stage of the search. To address these issues, this paper employs several strategies to improve SSA. Firstly, the population is initialized using Logistic Chaos Mapping [37] to expand the search range of the algorithm and enrich its population diversity. Then an adaptive method is used to adjust the proportion of vigilantes (SD) and the proportion of discoverers (PD) to adjust the global and local search capability of SSA according to the situation during the search process. Meanwhile, the Levy flight strategy [38] is introduced into the vigilante position update formula to improve SSA’s ability to jump out of the local optimum. Then, an elite learning strategy [39] is introduced to learn and utilize the information of historically optimal individuals to improve the search capability of the algorithm. Finally, an early-stopping strategy is added to the algorithm, that is, when the fitness value is not improved and the number of times reaches the early-stopping threshold in consecutive iterations, the algorithm will eventually stop iterating and output the optimal solution. The flowchart of the improved MSSA is shown in Fig. 1:

The pseudo-code is presented in Fig. 2. We have highlighted the parts that differ from the original SSA for ease of differentiation.

#### Use the logistic chaos mapping strategy to initialize the population

Chaos theory is a nonlinear theory and has good applications in random number generation. Many swarm intelligence optimization methods use chaos mapping as random number generators to initialize populations [40]. The original SSA algorithm uses a completely random population initialization, and there will be a problem of insufficient population diversity. Therefore, this paper introduces the logistic chaos mapping into SSA to initialize the population, so as to expand the search range of the algorithm and enrich its population diversity. Logistic chaos mapping is a nonlinear mapping defined on the [0,1] interval, and its formula is (5):

5 $$c_{n + 1} = \mu c_{n} (1 - c_{n} ),c_{n} \in [0,1],\mu \in R^{ + }$$

When using the logistic chaos mapping to initialize the population, it is necessary to generate a matrix $C$ with the same size as the formula (1), as shown in the formula (6), each element of which is generated by the formula (5), and finally through the formula (7) calculate the initial position of each individual. Initializing the population by means of logistic chaos mapping helps the algorithm to search globally and improves the possibility of finding the global optimal solution.

6 $$C = \left[ {\begin{array}{*{20}l} {c_{1,1} } & {c_{1,2} } & {...} & {c_{1,j} } \\ {c_{2,1} } & {c_{2,2} } & {...} & {c_{2,j} } \\ \vdots & \vdots & \vdots & \vdots \\ {c_{i,1} } & {c_{i,2} } & {...} & {c_{i,j} } \\ \end{array} } \right]$$

7 $$X_{i,j} = lb_{j} + (ub_{j} { - }lb_{j} {) } \times {\text{C}}_{i,j}$$

#### Adaptive adjustment parameter strategy

In the SSA in this paper, an adaptive parameter adjustment strategy is added, which can dynamically adjust the proportion of discoverers (PD) and the proportion of vigilantes (SD) during each iteration. The strategy takes the current number of iterations as input, and then adaptively adjusts the two key parameters, PD and SD, according to the search phase (early or late) of the algorithm.

In the initial stage of the search, a higher proportion of discoverers is set to enhance the global search capability of the algorithm, so that it is possible to find a better solution in the entire search space. Gradually reduce the proportion of discoverers as the search progresses. The setting of the proportion of vigilantes is opposite to discoverers, that is, a lower proportion of vigilantes is set at the beginning of the search, and the proportion of vigilantes is gradually increased in the later stage of the search. Doing so can improve the local search ability of SSA, so that more detailed searches can be carried out near the excellent solutions that have been found, and the accuracy of the solutions can be improved.

This adaptive adjustment parameter strategy fully balances the needs of global search and local search, helps to improve the performance of the algorithm in different search stages, and finds the optimal solution quickly.

#### Levy flight strategy

In the original SSA search process, the vigilante is mainly responsible for fine local search of the search space, that is, deep exploration near the current known good solutions to find possible better solutions. However, only relying on local search may make the algorithm fall into local optima, which limits the global search ability of the algorithm. Therefore, Levy flight strategy is introduced to update the position of the vigilante in SSA. First, the Mantegna method is used to generate the random step size of the Levy plane, which can be obtained from formula (8):

8 $$\begin{aligned} s & = \frac{u}{{\left| v \right|^{{\frac{1}{\beta }}} }} \\ u & \sim N(0,\delta_{u}^{2} ),v\sim N(0,\delta_{v}^{2} ) \\ \delta_{u} & = \left\{ {\frac{\Gamma (1 + \beta )\sin \pi \beta /2}{{\Gamma [(1 + \beta )/2]\beta \cdot 2^{(\beta - 1)/2} }}} \right\}^{{\frac{1}{\beta }}} ,\delta_{v} = 1 \\ \end{aligned}$$

where $\beta$ in formula (8) is a constant, and the position update formula of vigilante introduced into Levy flight strategy is changed from formula (4) to formula (9):

9 $$X_{i,j}^{t + 1} = \left\{ \begin{aligned} X_{best}^{t} + \beta \left| {X_{i,j}^{t} - X_{best}^{t} } \right| \cdot s,\quad f_{i} \ne f_{g} \hfill \\ X_{best}^{t} + k\left( {\frac{{X_{i,j}^{t} - X_{best}^{t} }}{{\left| {f_{i} - f_{w} } \right| + \varepsilon }}} \right),\quad f_{i} = f_{g} \hfill \\ \end{aligned} \right.$$

Levy flight is a random search behavior. This behavior is manifested as the probability of short-distance search and long-distance search interleaved. When the step of Levy flight is large, it can expand the breadth of the search, thereby increasing the possibility of global optimization. When the step size is small, it can enhance the accuracy of local search and improve the ability of local optimization. Thus, this diversity of Levy flights can conduct a more comprehensive search in the solution space.

#### Elite learning strategies

Using the strategy of elite learning, the purpose is to retain a certain proportion of the best individuals in each iteration, and these excellent individuals will be introduced into the next generation population. Specifically, we first calculate the number of elite individuals ($elite\_size$), which is determined by the product of the population size ($N$) and the elite rate. Then, sort according to the size of the fitness value, and select $elite\_size$ best individuals as elite individuals. Then, these elite individuals are merged with the current population to form a new population, and the fitness values of the new population are also sorted. Finally, the same number of individuals with the same size as the original population are selected from the new population according to the sorting result as the next generation population.

The application of this strategy ensures the survival and inheritance of excellent individuals, and also allows new individuals to enter the population, thus ensuring the diversity of the population and helping the algorithm to jump out of the local optimum and improve the global search capability.

#### Early stopping strategy

In the model, an early stopping strategy is also added, that is, an early stopping threshold is first set, and when the fitness value is not improved and the number of times reaches the early stopping threshold, the iteration will be stopped. Generally, the early stopping threshold is set to half the number of iterations, which can effectively save computing resources and improve optimization efficiency.

### Using MSSA to optimize XGBoost parameters and feature selection

**Fig. 3:**
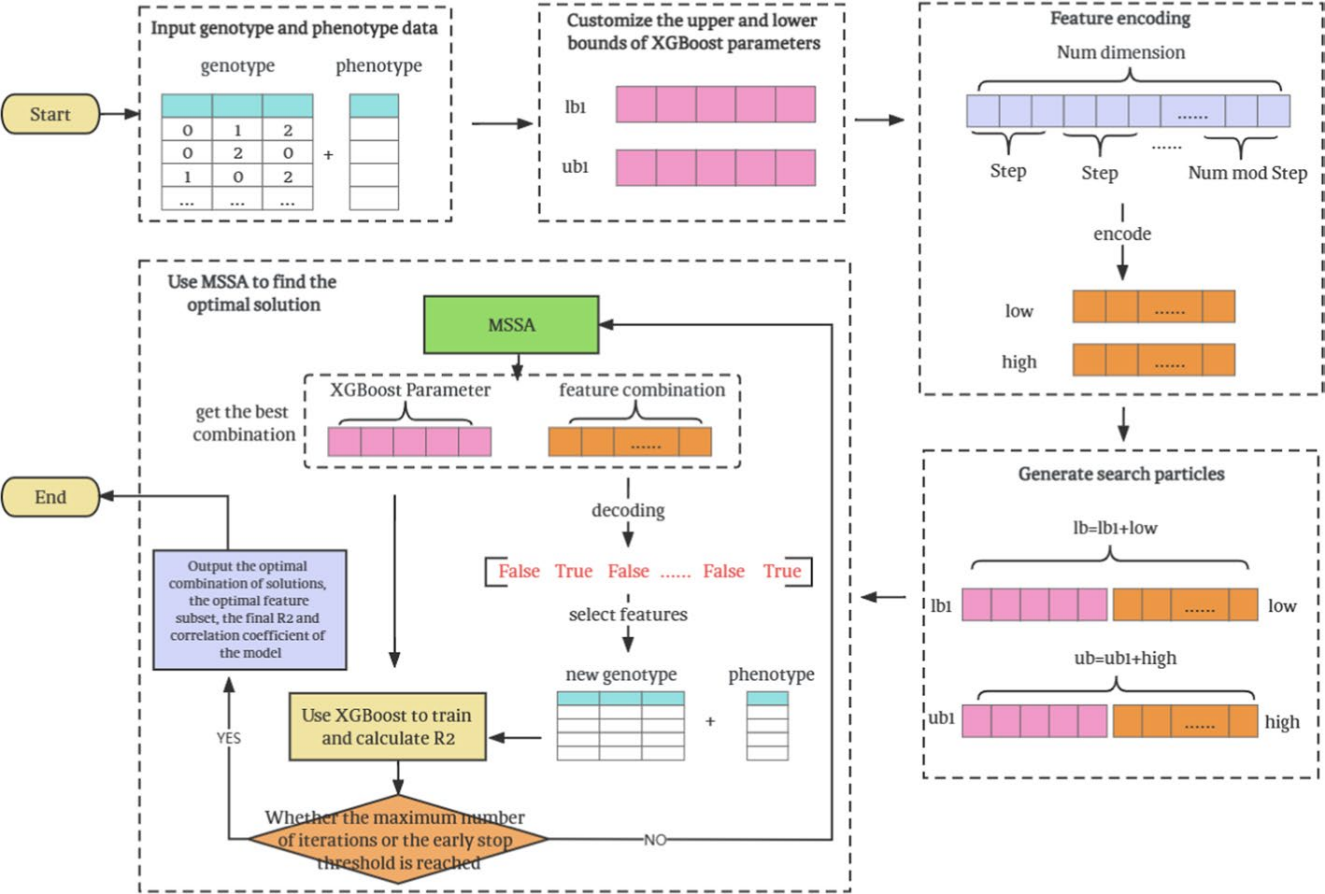
Overall framework of MSXFGP

The MSXFGP method implements the simultaneous optimization of XGBoost parameters and feature selection using MSSA, and its overall framework is shown in Fig. 3:

The specific steps of MSXFGP are explained as follows:

*Step 1* Input genotype and phenotype data;

*Step 2* Customize the upper and lower bound lists *ub1, lb1* of the five parameters that need to be optimized in XGBoost;

*Step 3* Feature encoding, generate the minimum selection number list *low* and the maximum selection number list *high* of the feature grouping, the specific process of feature encoding is described in Section “Feature encoding and decoding”;

*Step 4* Generate search particles according to Steps 2 and 3, that is, the upper and lower bound lists *ub* and *lb* that need to be optimized;

*Step 5* Use the MSSA algorithm to optimize the search particles;

*Step 6* Decode the list of selection numbers of the searched feature groups, and perform feature selection. For the decoding process, see the description in Section “Feature encoding and decoding”;

*Step 7* According to the searched XGBoost parameters and the genotype data after feature selection, input the XGBoost model for training, and calculate the fitness R^2^ coefficient;

*Step 8* Determine whether the maximum number of iterations or the early stop threshold is reached. If so, stop using MSSA to search, output and save the optimal search particle combination, the optimal feature subset, the final R^2^ and correlation coefficient results of the model, and end the algorithm process; otherwise return to step 5.

The following is a detailed description of some of the details:

#### Feature encoding and decoding

The input of this process is two parameters *Num* and *Step*, where *Num* represents the total number of features, and *Step* represents the step size when performing feature selection. Take feature *Num* = 25, step size *Step* = 10 as an example to describe the process of feature encoding and decoding in detail:

1. Calculate the group length list (*group_length*): This is the number of features for each group calculated based on the total number of features *Num* and the *step* size Step, that is, *group_length* = *[Step,Step,…,Num mod Step]*, where *mod* is a remainder operation. For example, the number of features is 25, and the input with a step size of 10 will be divided into three groups. The first group has 10 features, the second group also has 10 features, and the third group has 5 features. The *group_length* is [10, 10, 5]. This value reflects the segmentation strategy for the feature space, where each group is an independent search space.
2. Calculation of the minimum selection list of groups (*low*): Each value in it specifies the minimum number of features that must be selected for each group. Except for the last group, the minimum selection number of other groups is 0, which means that these groups can not select any features; and the minimum selection number of the last group is 1, ensuring that at least one feature is selected. At this time list *low* is [0, 0, 1].
3. Calculate the group maximum selection list (*high*): This is the maximum number of feature selections calculated according to the length of each group. This value is obtained by converting the length of each subgroup to a corresponding all “1” binary string and then to a decimal integer. This list defines the maximum number of features that can be selected for each group, thereby limiting the size of each independent search space. For example, the length of the group is 10, first converted to a binary string of “111111111111”, and then converted to a decimal value of 1023. If the length of the group is 5, then the binary string is “11111”, converted to decimal 31, and finally the *high* is [1023, 1023, 31].
4. Feature decoding: An integer list is obtained through a search algorithm between the minimum selection list (low) and the maximum selection list (high), where each integer represents the selection of a group. Convert this integer list to a binary string first, and then convert it to a Boolean array, where the length of the Boolean array is equal to the total number of features Num, and "True" in the final Boolean array means that the feature is selected, and "False" means that it is not selected. For example, if the result after searching is [3, 5, 2], first convert each integer value into binary, get the second list [0000000011, 0000000101, 00010], and then convert it into a Boolean array
  “[False, False, False, False, False, False, False, False, True, True, False, False, False, False, False, False, False, True, False, True, False, False, False, True, False]”, then the number of feature columns finally selected is 9, 10, 18, 20, 24.

#### Search particle design

**Fig. 4:**
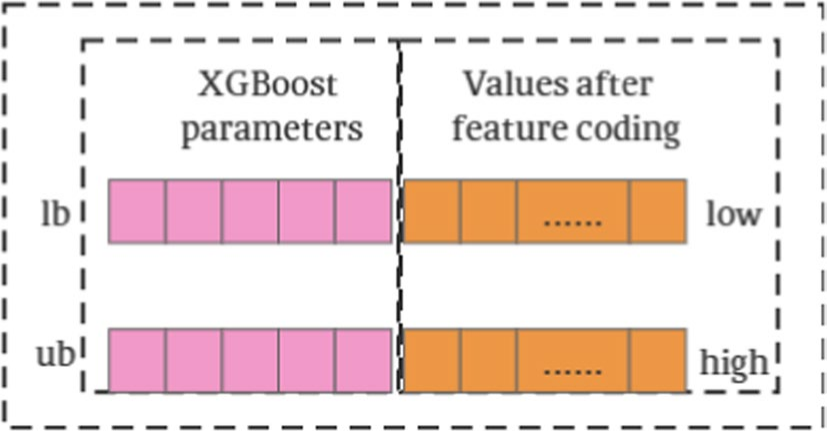
The range of search particles

When constructing the XGBoost regression model, several key parameters need to be set, and this study focuses on five parameters that need to be optimized: the learning rate (eta), the maximum depth of the tree (max_depth), the minimum weight of the child nodes (min_child_weight), the proportion of the sample subsamples, and the proportion of the column sample subsamples (colsample_bytree). The traditional strategy is usually to optimize these parameters based on all the features first, followed by feature selection, but this may result in some features that are critical to the XGBoost model being missed during the feature selection process. Another strategy is to perform feature selection first and then optimize the parameters, but for a large number of features, this can make the feature selection process extremely time-consuming. Regardless of the independent optimization strategy, it may lead to a decrease in model accuracy. Therefore, in this paper, we adopt an approach that performs both XGBoost parameter optimization and feature selection to improve the accuracy of the regression model and significantly reduce the computational time cost. In this method, the dimension searched by each searching individual consists of two parts: the first part is the search range of the five parameters “eta, max_depth, min_child_weight, subsample, colsample_bytree”; the second part is the integer value between the list of the smallest number of choices (low) and the list of the largest number of choices (high) in Section “Feature encoding and decoding”. The latter part is the integer value between the minimum selection list (low) and the maximum selection list (high) in Section “Feature encoding and decoding”. The range of search particles is shown in Fig. 4:

#### Design of the fitness function

In this study, a fitness function is constructed whose input is a search particle optimized by MSSA. The first five elements of this particle represent the five key parameters of the XGBoost model (eta, max_depth, min_child_weight, subsample, and colsample_bytree), whereas the remaining elements undergo the feature decoding process in Section “Feature encoding and decoding”, which is used to perform the feature selection to produce new genotype data. Based on the parameters after MSSA optimization and the genotype data after feature selection, the XGBoost regression model is trained, and finally, the R^2^ score is calculated as a fitness metric to measure the performance of the model, and the formula for R^2^ is as follows:

10 $$R^{2} = 1 - \frac{{\sum {(y - \hat{y})^{2} } }}{{\sum {(y - \overline{y})^{2} } }}$$

where $\hat{y}$ represents the phenotype value predicted by the model, $\mathop y\limits^{{}}$ represents the real phenotype value, and $\overline{y}$ represents the average value of the real phenotype value.

## Results and discussion

### Limitations of using only the Pearson correlation for evaluation

The Pearson correlation coefficient measures the linear correlation between two variables, with values ranging from − 1 to 1, with 1 indicating a perfect positive correlation, − 1 indicating a perfect negative correlation, and 0 indicating no correlation. The correlation coefficient is the correlation between the predicted and actual values of the phenotype, and the larger the value, the better the model's prediction matches the actual results. The formula for calculating the Pearson correlation coefficient is as follows:

11 $$cor = \frac{{Cov(y,\hat{y})}}{{\sigma_{y} \sigma_{{\hat{y}}} }}$$

where $\hat{y}$ represents the phenotypic value predicted by the model and $\mathop y\limits^{{}}$ represents the true phenotypic value.

The coefficient of determination (R^2^), also known as the goodness of fit, is a commonly used statistical indicator in regression analysis, with the formula shown in (10), which is used to evaluate how well the model explains the sample observations, with a range of values generally between 0 and 1, but the value may be negative when the model does not fit the data at all. The closer the value is to 1, the better the model fits the data.

**Fig. 5:**
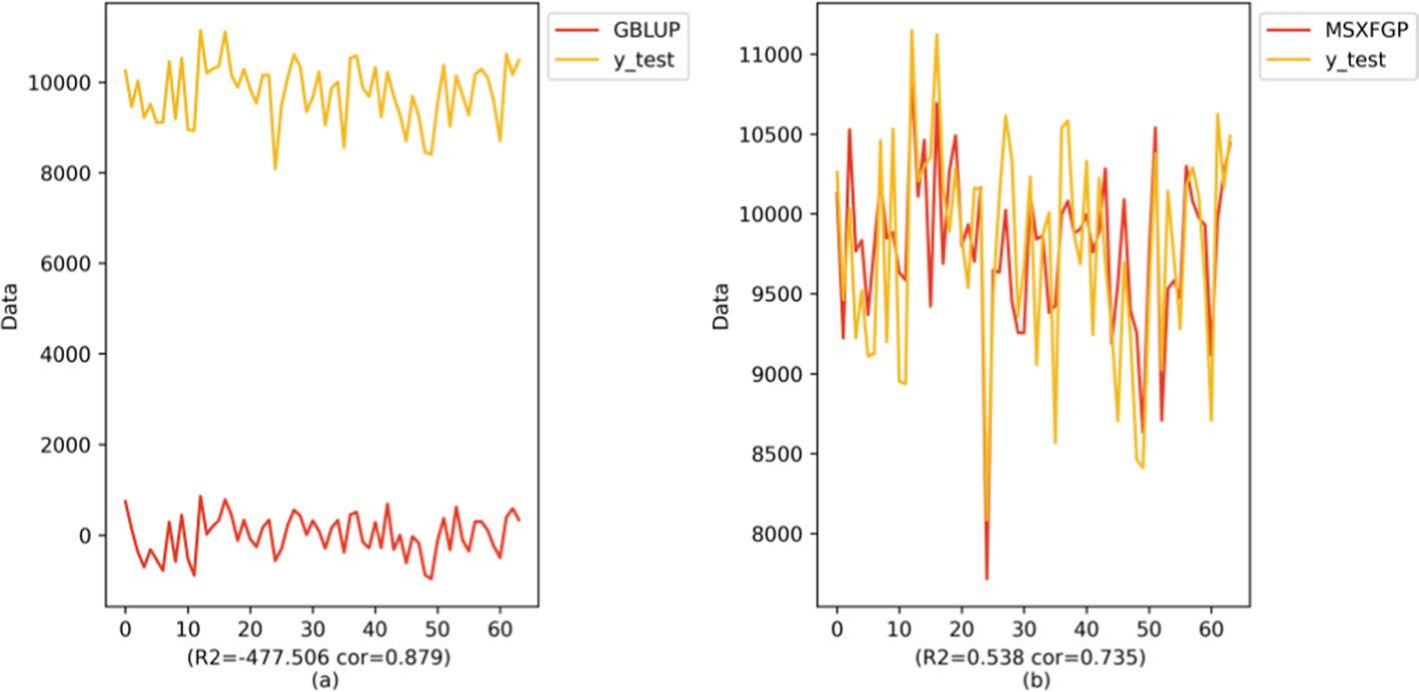
Comparison of predicted and true values of GBLUP and MSXFGP models

In this section, we take the Japonica dataset as an example. When using MSXFGP, we adopt a common data set division strategy, select 80% of the samples in the data set as the training set, and select 20% of the samples as the test set for the model training and validation. However, since GBLUP directly calculates the breeding value, only a test set of 20% samples is used for comparison with MSXFGP. The experimental results are shown in Fig. 5:

The results using GBLUP show that the coefficient of determination (R^2^) and Pearson’s correlation coefficient (cor) derived on the test set are − 477.506 and 0.879. Although the Pearson correlation coefficient is higher, showing a stronger linear relationship between the predicted and real values, the coefficient of determination is negative, which is significantly deviating from the theoretical value (should be in the range of 0–1), indicating that the model prediction is significantly different from the actual results are significantly different. The results of the experiments using MSXFGP show that the coefficient of determination (R^2^) and Pearson’s correlation coefficient (cor) of the model on the test set are 0.538 and 0.735, respectively. Although the correlation coefficient is not as high as GBLUP, the R^2^ value is within a reasonable range, which indicates that the model can explain at least part of the data variance, that is, the prediction results of the model are reasonable and credible. At the same time, the correlation coefficient of 0.735 also reflects that there is a certain linear connection between the model’s predicted values and the actual results.

In Fig. 5, we can clearly see that there is better agreement between the predicted results and the true values of MSXFGP compared to GBLUP. Although the Pearson correlation coefficient is often used as a metric to assess the linear relationship between model predictions and actual results, there are inherent limitations in relying on this coefficient alone for model evaluation. Firstly, the Pearson correlation coefficient only measures the strength of the linear relationship between predicted and actual results and does not accurately reflect the accuracy of the predictions. If the predicted results deviate from the actual results to a large extent, the correlation coefficient may be high even if it maintains a strong linear relationship, but this does not indicate that the prediction is accurate. In addition, the Pearson correlation coefficient does not deal well with non-linear relationships. If there is a complex nonlinear relationship between the predicted results and the actual results, it is difficult to fully and accurately evaluate the model performance only by the Pearson correlation coefficient.

Therefore, this paper uses the coefficient of determination (R^2^) and the Pearson correlation coefficient (cor) as evaluation indicators to double-evaluate the prediction accuracy of the model and the relationship between the prediction results and the actual results. This assessment can reflect the prediction effect of the model more comprehensively and accurately, which helps us understand and optimize the model from a more comprehensive perspective.

### Comparison between MSXFGP and the method before improvement

**Fig. 6:**
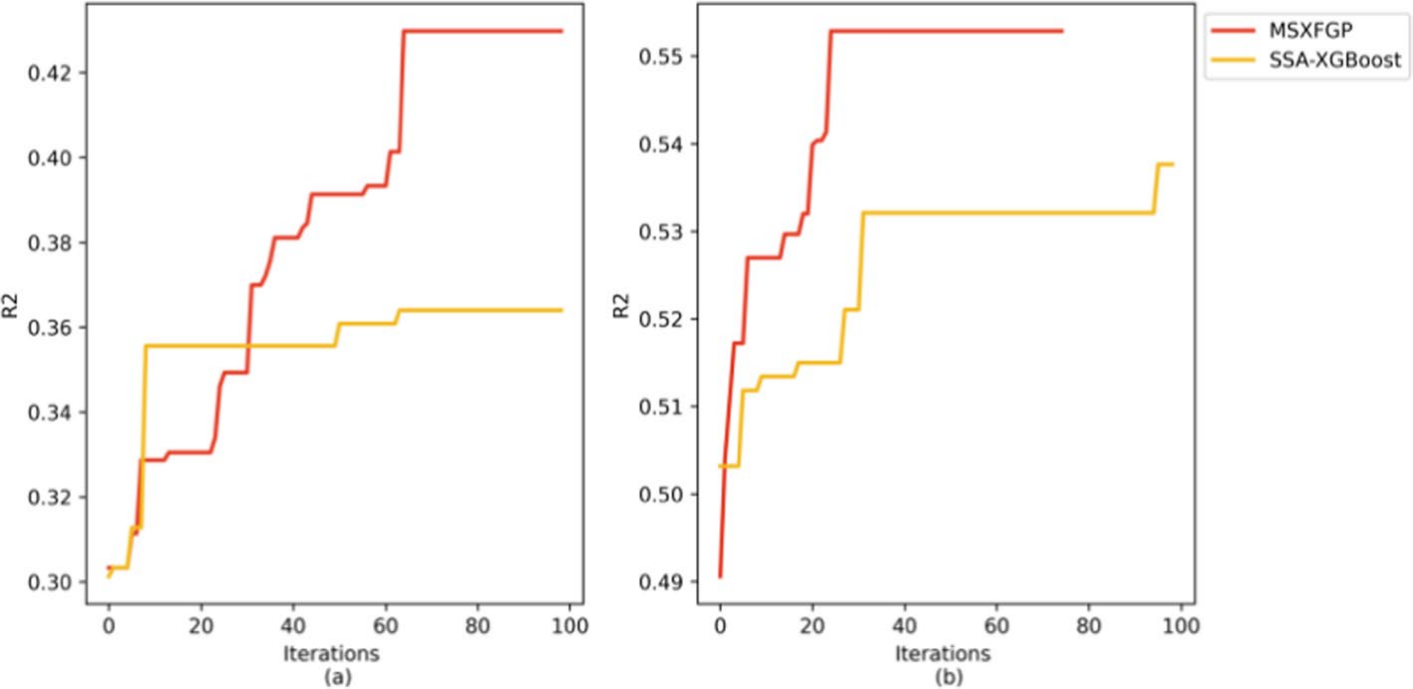
Comparison of R^2^ convergence between MSXFGP and the method before improvement

In this section, in order to fully evaluate and validate the effectiveness of MSXFGP, we conduct a comparative study with the unimproved SSA algorithm, using two independent potato datasets as experimental subjects. To further improve the stability and accuracy of the experimental results, this subsection employs a five-fold cross-validation strategy to minimize the risk of overfitting as much as possible. As shown in Fig. 6, compared with the algorithm before improvement, MSXFGP has significantly improved the performance of the coefficient of determination (R^2^). This fully demonstrates the effectiveness of our improvement strategy in improving the prediction accuracy of the model. The improved strategy not only effectively reduces the possibility of the model falling into a local optimal solution during the solution process, but also successfully promotes the prediction results to the direction of the global optimal solution. In addition, as shown in Fig. 6b, we introduce an early stopping strategy during model training, which allows us to terminate training early after the model reaches a certain preset performance standard. In this way, while ensuring prediction accuracy, the time consumption of training is reduced, and the practicability of the model is improved.

### Prediction accuracy of MSXFGP and comparison with other methods

**Fig. 7:**
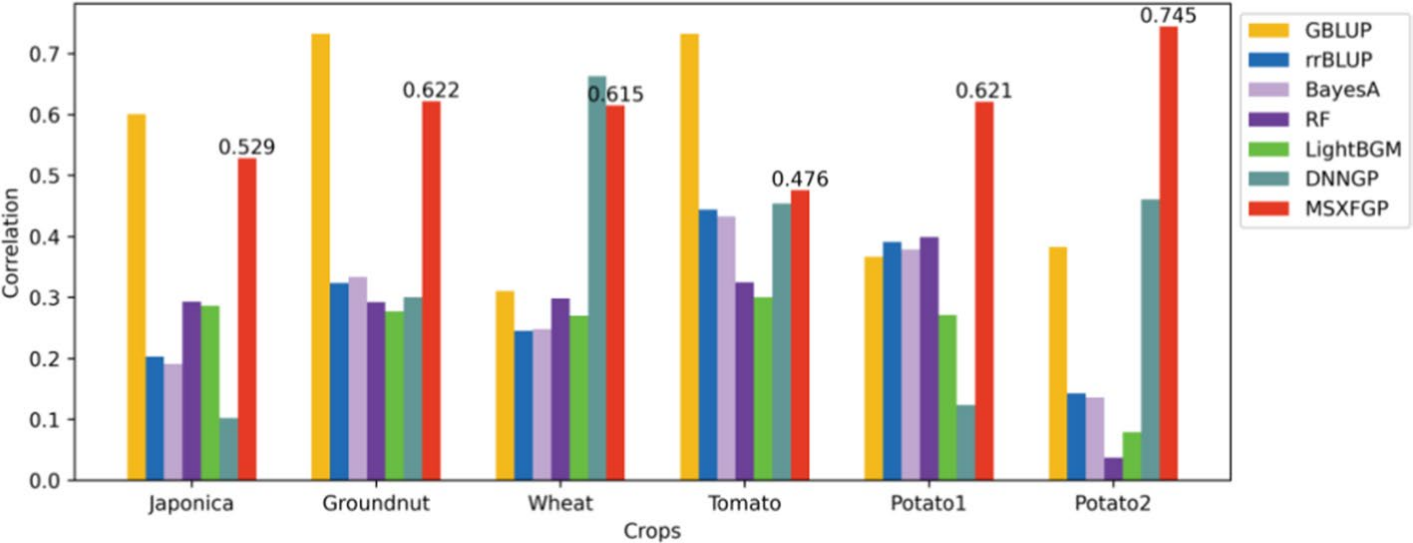
Prediction results of MSXFGP and comparison with other methods (Pearson correlation coefficient)

**Fig. 8:**
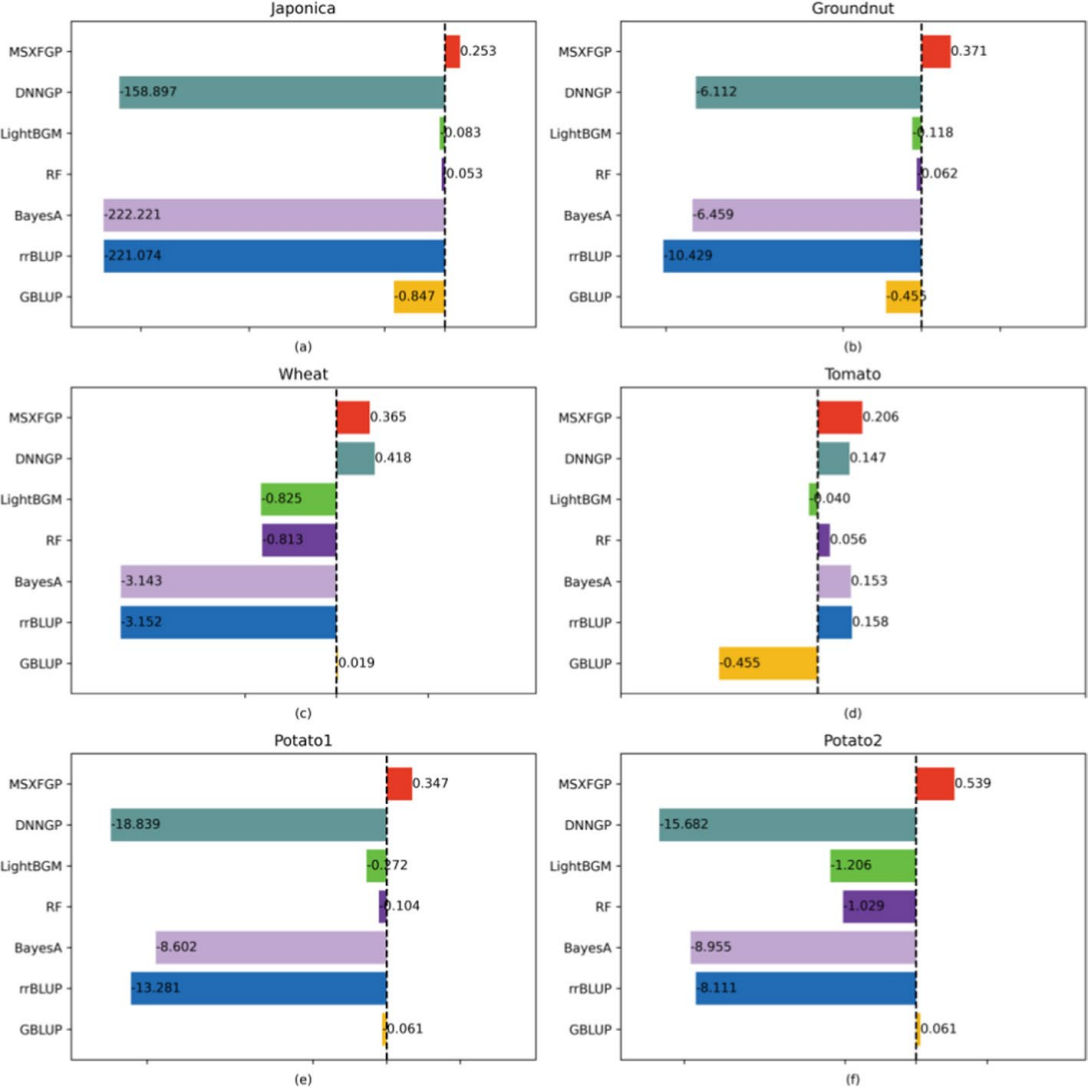
Prediction results of MSXFGP and comparison with other methods (R^2^)

In this paper, MSXFGP continued to be evaluated in comparison with several different genome selection methods on different crop datasets. Where Fig. 7 shows the results based on the Pearson correlation coefficient (cor) as an evaluation metric and Fig. 8 shows the results based on the coefficient of determination (R^2^) as an evaluation metric.

In Fig. 7, from the Pearson correlation coefficients, the results of GBLUP are better than MSXFGP in only three datasets. However, from the combined view of Figs. 7 and 8, our MSXFGP outperforms or is comparable to the results of the other methods in all datasets. It is worth saying that MSXFGP has R^2^ values greater than 0 on all six datasets, which indicates that our model performs well in fitting the data. Especially on the potato2 dataset, after ten-fold cross-validation, the R^2^ value of MSXFGP reaches 0.539, which is much higher than other methods. Also, its Pearson correlation coefficient is generally high, which further proves that the prediction results of our model are in high agreement with the actual results.

Although the results of DNNGP are better than MSXFGP on the wheat data set, it should be noted that the tuning of hyperparameters by DNNGP depends on rich experience and practice. In fact, the author of DNNGP only provided the optimal hyperparameter settings tuned on the wheat and tomato datasets in the paper, and the rest of the datasets were not used in their paper, so only the default parameters were used in this paper to train other datasets. This also indirectly proves the superiority of the MSXFGP model, as it can perform well on multiple datasets without complex hyperparameter tuning.

To ensure the reliability of the method results, we conducted relevant statistical analysis, with the specific data presented in Table 1.

**Table 1:** Statistical analysis of MSXFGP results

Crop	*p*-value Range	Mean *p*-value	R^2^ confidence Interval (95%)	Mean R^2^
Japonica	(0.000289,0.009727)	0.00333151	(0.221, 0.286)	0.253
Groundnut	(1.01e-08,0.028108)	0.003766639	(0.262, 0.477)	0.371
wheat	(1.03e-10,0.001188)	0.000119112	(0.284, 0.447)	0.365
tomato	(0.000105,0.063222)	0.01605068	(0.121, 0.291)	0.206
potato1	(1.66e-07,0.013089)	0.003673015	(0.266, 0.428)	0.347
potato2	(3.36e-18,1.80e-08)	1.88E-09	(0.466, 0.612)	0.539

First, the *p*-value, a core metric in statistics, serves as an indicator to evaluate the likelihood of observing results under the null hypothesis. In this study, the null hypothesis posits that there is no significant correlation between the predicted and actual values. Using ten-fold cross-validation, we calculated the *p*-value for each validation separately. Both the range and the mean of the *p*-values across the ten validations were significantly below 0.05. This statistically indicates a significant correlation between the predicted and actual values, providing grounds to reject the null hypothesis.

Secondly, not only did we calculate the R^2^ values for each cross-validation, but we also further determined the confidence intervals and their means of these R^2^ values at the 95% confidence level. As evident from Table 1, the mean R^2^ values for each dataset consistently fall within their 95% confidence intervals, further reinforcing the reliability of our method’s results.

### MSXFGP tool

Finally, to make it easier for breeders to utilize the MSXFGP method, we developed it into a user-friendly tool using the Python programming language. This tool accepts genotype and phenotype data as input. The genotype data can either be in the encoded format of 0, 1, 2, 3 or be numerical data reduced through PCA. Currently, the phenotype data only supports numerical data, that is, quantitative traits, and does not support qualitative traits. The accepted data format is CSV. The tool’s output files include the R^2^ historical values during model training, the final optimized XGBoost parameters, and the genotype file after feature selection. With the optimized parameters and the new genotype file, users can easily train an XGBoost model to predict their data, accomplishing the genomic selection task. We have open-sourced this tool on GitHub. For more specific usage methods, users can refer to and learn from https://github.com/DIBreeding/MSXFGP.

### Discussion on MSXFGP parameters

When using the MSXFGP tool, there are several key parameters that users need to set themselves: population size, number of cross-validation folds, and number of iterations. Using the potato2 dataset as an example, we conducted experiments with population sizes of 10, 30, 50, and 100, cross-validation fold counts of 3, 4, 5, and 10, and 50 iterations. We recorded the final R^2^, method runtime, and the significance level (*p*_value) of the correlation between predicted values and actual values. The results are shown in Table 2.

**Table 2:** The results of different parameters of MSXFGP

Population size	Evaluation indicator	Threefold	Fourfold	Fivefold	Tenfold
10	R^2^	0.522	0.526	0.521	0.520
	Time (s)	263	609	614	564
	*p*_value	2.28E-36	6.20E-26	4.66E-20	2.08E-08
30	R^2^	0.509	0.546	0.534	0.521
	Time (s)	454	1247	1604	1471
	*p*_value	7.43E-35	1.02E-27	1.02E-20	5.68E-09
50	R^2^	0.527	0.534	0.545	0.524
	Time (s)	1046	2054	2308	2626
	*p*_value	1.66E-36	1.32E-26	2.50E-20	1.52E-08
100	R^2^	0.533	0.532	0.535	0.546
	Time (s)	3186	4294	3394	4917
	*p*_value	1.13E-34	1.84E-28	2.36E-20	1.81E-08

The results indicate that for all parameter combinations, the *p*_value is far below 0.05, suggesting that the model’s results under different population sizes and different cross-validation fold numbers are significant. Meanwhile, as the population size gradually increases, although R^2^ shows some fluctuations, it generally presents an upward trend. In addition, with the same number of cross-validation folds, computation time also increases as the population size grows. It’s worth noting that computation duration is not only related to parameter settings but also closely tied to the computer’s hardware configuration and data scale. Regarding the number of iterations, Fig. 8f shows that when the population size is 50, the number of iterations is 100, and using ten-fold cross-validation, the R^2^ for the potato2 dataset is 0.539. However, in Table 2, when the number of iterations is 50, the corresponding R^2^ is 0.524. This suggests that as the number of iterations increases, R^2^ also shows a rising trend.

In summary, different parameter definitions yield different results. Larger population sizes and more iterations might lead to better prediction result but would significantly increase computation time. Higher cross-validation fold numbers can enhance the reliability of results but also add to computational load. These problems are also our next optimization direction.To ensure the reliability of the results while minimizing time consumption, users might consider using MSXFGP with parameter settings of five-fold cross-validation, a population size of 50, and a maximum iteration count of 50.This represents a trade-off between computational efficiency and the level of model optimization. However, for researchers willing to invest more time in model training and optimization, they can definitely consider adjusting these parameters for a broader range of optimization, potentially yielding more valuable research outcomes.

## Conclusion

In this study, we proposed a genome prediction method MSXFGP based on a multi-strategy improved SSA with simultaneous optimization of XGBoost parameters and feature selection. Through testing on six datasets, the MSXFGP model outperforms or is comparable to the current mainstream model prediction in prediction accuracy, and it shows good prediction results under both the Pearson correlation coefficient and the coefficient of determination R^2^ as the evaluation metrics. In addition, we also provide a user-friendly Python tool to reduce the difficulty of using the model for breeders. In conclusion, MSXFGP is expected to be a promising and practical genome selection model to help breeders achieve better results in practical work.

In the future, we plan to further optimize MSXFGP. Considering the potential demonstrated by deep learning in multiple bioinformatics challenges, we aim to combine it with our current methods to enhance the speed and accuracy of genomic predictions. Additionally, we will explore how to integrate environmental data to bolster the model's predictive performance in practical applications. Lastly, we plan to collaborate with breeding experts to incorporate MSXFGP into the intelligent breeding systems under development, hoping to offer more possibilities and potential value to modern agricultural production.

## Data Availability

Code and data are placed at https://github.com/DIBreeding/MSXFGP. The original data can be located in the respective references.
